# On the intercultural sensitivity of university students in multicultural regions: A case study in Macao

**DOI:** 10.3389/fpsyg.2023.1090775

**Published:** 2023-02-23

**Authors:** Hong Chen, Bo Hu

**Affiliations:** ^1^Faculty of International Tourism and Management, City University of Macau, Macao, Macao SAR, China; ^2^University International College, Macau University of Science and Technology, Macao, Macao SAR, China

**Keywords:** intercultural sensitivity, intercultural communication, university students, multicultural regions, Macao

## Abstract

Intercultural sensitivity has been regarded as a very important ability necessary for living in multicultural countries and regions. In this research, the quantitative method was used to explore the intercultural sensitivity of university students in Macao, a typical multicultural region in China. By adopting the Intercultural Sensitivity Scale developed by Chen and Starosta and a sociodemographic questionnaire designed by the authors, this study investigated the intercultural sensitivity level of university students in Macao, and explored whether there were any significant differences in student groups in terms of gender, grade, program (language of instruction adopted in a major), courses related to intercultural communication, overseas experience, and foreign language proficiency. The findings of the study on a sample of 375 participants showed that university students in Macao had a relatively high intercultural sensitivity level. There were significant differences in different student groups in terms of gender, grade, courses related to intercultural communication, and foreign language proficiency. This study has implications for both educational policymakers and educators and can ultimately help university students in a multicultural environment improve their intercultural sensitivity and sustainable development.

## 1. Introduction

Due to the globalization and internationalization of the whole world, intercultural communication competence has become increasingly significant (Jackson, 2011; Deardorff and Jones, 2012; Wang et al., 2017). In a multicultural country or region, it is even more urgent than before to strengthen the cultivation of international talents with intercultural capabilities.

Intercultural communication competence is a complex conceptual system that has been defined in various ways, as a combination of three elements including motivation, knowledge, and skills (Spitzberg and Cupach, 1984), as “the ability to encode and decode meanings in matches that correspond to the meanings held in the other communicator’s repository” (Beamer, 1973), as “the ability to think and act in interculturally appropriate ways” (Hammer et al., 2003), as a combination of affective, cognitive, and behavioral dimensions (Chen and Starosta, 1996; Ting-Toomey, 1999; Sptizber, 2000; Bennett, 2001; Fritz et al., 2001; Müller and Gelbrich, 2001; Graf, 2004), as “the cultural, social, and psychological knowledge that groups of people have about appropriate communication patterns and their ability to use this knowledge *in situ* [*sic*]” (Witteborn, 2003), and as a person’s ability to receive and send positive emotional signals before, during and after intercultural interaction (Fritz et al., 2005), etc.

To elaborate on the affective, cognitive, and behavioral dimensions of intercultural competence, Chen and Starosta (1996) proposed three corresponding concepts, including intercultural sensitivity, intercultural awareness, and intercultural adroitness. Among them, intercultural sensitivity is the affective component of intercultural competence. Intercultural sensitivity is a prerequisite for successful intercultural communication (Chen, 1997), and it has become a strong demand for living harmoniously and meaningfully in today’s pluralistic world (Chen, 1997). Greater intercultural sensitivity is associated with greater potential for exercising intercultural competence (Hammer et al., 2003). In other words, only with a high intercultural sensitivity can we recognize differences in a multicultural environment, respect them, and conduct appropriate and effective intercultural communication.

Among the multicultural regions in the world, Macao is a city of 400 years of integration of Eastern and Western cultures (Li, 2017). Leased to Portugal in 1557, Macao became a trading port of southern China and opened to the world. Since the opening of the port, people of different nationalities came to Macao and settled down, and their cultural traditions permeated the life of the city. The interaction between Eastern and Western civilizations has created a unique Macao culture, where diverse ethnic groups, multilingual landscapes, various religious beliefs, different architectural styles, and varied customs have integrated here harmoniously.

Multiculturalism of Macao is firstly manifested in its multicultural population. According to the latest data collected by the Statistics and Census Service of Macao SAR (DSEC), in the third quarter of 2022, Macao has a total population of 671,900, of which 153,841 are non-resident employees. The non-resident employees come from Mainland China (69.3%), the Philippines (16%), Vietnam (5.1%), Indonesia (2.8%), Nepal (1.8%), Hong Kong (1.7%), and other countries and areas (3.3%; DSEC, 2022).

Diverse population has enabled Macao to be a multilingual city. The language landscape of Macao has been summarized by Chen and Li (2019) as “two characters (traditional and simplified), three written languages (Chinese, Portuguese, and English) and four spoken languages (Mandarin, Cantonese, Portuguese, and English)”. Among the written languages, Chinese and Portuguese are the official languages of Macao. While as for the spoken languages, Cantonese is the most widely used one in Macao. Since many of the residents are immigrants from Fujian Province, Hokkien is also widely used in their daily life (Yan, 2016). In addition, employees from the Philippines, Vietnam, Indonesia, and Nepal like to use their native language when communicating with their country fellows.

The fascinating blending of Eastern and Western cultures in Macao is also reflected in its unique multi-religious cultural landscape. Buddhism, Taoism, Catholicism, Christianity, Islam, and other Eastern and Western religions coexist harmoniously. Connected with the diversified religions, the juxtaposition of Western and Chinese architectural heritage sites in the city can be seen here and there. Among the buildings which belong to Historic Center of Macao (China’s 31st World Cultural Heritage), there are many Chinese temples (such as A-ma Temple, Kuantai Temple, and Na Tcha Temple) and Western churches (such as The Cathedral, St. Augustine’s Church, St. Lawrence Church, St. Anthony’s Church). Na Tcha Temple even stands side by side with The Ruins of the St. Paul’s (the façade of what was originally the Church of Mater Dei built in 1602–1640 and the ruins of St. Paul’s College).

As for its varied customs, Macao boasts a unique festival culture, including traditional Chinese celebrations such as Spring Festival, Dragon Boat Festival, Mid-autumn Festival, Double Ninth Festival, as well as the important Western festivals of Easter, Christmas, All Soul’s Day, Procession of our Lady of Fátima, and Feast of Immaculate Conception. Furthermore, with cultural integration, Macao cuisine has become the most inclusive food spectrum. Designated as UNESCO Creative City of Gastronomy in 2017, Macao not only offers Cantonese cuisine and Portuguese cuisine, but also offers all kinds of cuisines from other parts of China, as well as cuisines from other countries like Japan, Korea, Thailand, India, Brazil, Italy, and France.

The integration of Chinese and Western cultures in Macao laid down good foundations for cultivating talents with international vision. University students in Macao not only live in a multicultural social environment, but also study on a multicultural campus. Many academic and research staff have either studied or worked in overseas universities and more and more non-local students come to study in Macao. According to DSES, in the 2019–2020 academic year, non-resident teachers in higher education in Macao came from five continents, accounting for 36.57% of the total number of teachers, and the number of registered non-local students accounted for 52.8% of the total number (DSEDJ, 2020). The programs in universities are conducted in Chinese, English, and Portuguese. Universities in Macao have established multiple forms of partnerships with more than 200 universities in Europe, America, Australia, Mainland China, Taiwan, and Hong Kong in educational programs, academic research, personnel training, student exchanges, and other areas. Students here are surrounded by a dense multicultural atmosphere.

As a famous international tourism city, Macao is committed to building a World Centre of Tourism and Leisure, cultivating talents with intercultural communication competence and international vision becomes urgent. With the increase of multinational companies, the increase of immigration, the emergence of foreign students, the joining of foreign employees, and the development of international conferences and large-scale international events, intercultural communication has become the norm, and cultivating talents with intercultural communication skills and international vision has become an urgent task of development of Macao. The first step of this task is to examine the current intercultural communication competence of Macao university students. Since intercultural sensitivity is a prerequisite for successful intercultural communication (Chen, 1997), the intercultural sensitivity of university students in Macao should be examined first, which is the core of this research.

### 1.1. Literature review

Intercultural sensitivity has been described for many decades. The research made by Bronfenbrener, Harding, and Gallway in 1958 is one of the earliest studies concerning sensitivity (Chen and Starosta, 1997). It indicates that intercultural sensitivity is similar to interpersonal sensitivity (sensitivity to individual differences). Bennett (1984) put forward the concept of intercultural sensitivity as a continuum in which one can move from ethnocentric stages to ethno-relative stages affectively, cognitively, and behaviorally. Pruegger and Rogers (1993) hold the view that intercultural sensitivity is the evaluation and tolerance of different cultures. Chen and Starosta (1997) put forward that intercultural sensitivity is the affective aspect of intercultural communication competence and defined it as “an individual’s ability to develop a positive emotion toward understanding and appreciating cultural differences that promote appropriate and effective behavior in intercultural communication.” Through further research, Chen and Starosta (2000) emphasized that to promote intercultural competence, interculturally sensitive people must possess self-esteem, self-monitoring, open-mindedness, empathy, interaction involvement, and non-judgment. Hammer et al. (2003) proposed that intercultural sensitivity refers to the ability to discriminate and experience the relevant cultural differences. Sinicrope et al. (2007) pointed out that intercultural sensitivity is an individual’s “ability to step beyond [his/her] own culture and function with other individuals from linguistically and culturally diverse backgrounds.” Chen (2009) defined intercultural sensitivity as the positive emotional ability of individuals to understand and appreciate cultural differences and promote appropriate and effective intercultural communication. Moore-Jones (2018) held the view that intercultural sensitivity mainly exists within individuals and is related to the evolution of their attitudes toward cultural differences, rather than constituting a specific expression of their behavior. It helps to develop an ability in the affective dimension, enabling people to understand the differences between their own culture and others, thereby placing themselves in the position of others and perceiving and understanding the world in different ways.

Intercultural sensitivity research has experienced two stages: theoretical model construction and empirical research. In the field of applied linguistics and language education, there are currently 4 influential scales: (1) Bhawuk and Brislin (1992) created the Intercultural Sensitivity Inventory (ICI) to measure the intercultural sensitivity in both individualist and collectivist cultures. (2) Cushner (1986) developed the Inventory of Cross-cultural Sensitivity to measure people’s sensitivity to different cultures. The scale includes cultural integration, behavioral response, intellectual interaction, attitudes toward others, and empathy. (3) Hammer and Bennett (1998), based on cognitive psychology and constructivist theory, constructed the Intercultural Development Inventory (IDI), which has been used to measure people’s orientations toward cultural differences, including denial, defense, minimization, acceptance, adaptation, and integration. The scale has been widely used in the United States, Europe, and Asia. However, the fee for using this instrument (about US$1,500) has deterred many researchers. (4) Chen and Starosta (2000) believed that intercultural sensitivity is the emotional performance of intercultural communicative competence and developed the Intercultural Sensitivity Scale (ISS). The scale includes 5 dimensions: intercultural engagement, respect of cultural difference, interaction confidence, interaction enjoyment, and interaction attentiveness.

As one of the intercultural sensitivity testing tools with high reliability (Cronbach’s *α* = 0.88), the ISS developed by Chen and Starosta (2000) has been widely adopted in assessing people’s intercultural sensitivity in many countries, such as China, Thailand, Korea, Malaysia, Iran, Germany, United States, Chile, and Spain. The research respondents included students of different educational levels, English teachers, foreign language teaching assistants of Fulbright projects, foreign trade talents, employees of transnational companies, company boards, etc. The empirical research mainly covered surveys on intercultural sensitivity levels, comparison of intercultural sensitivity levels among different groups, psychometric testing of the ISS scale, correlation studies on factors affecting intercultural sensitivity, and the relationship between intercultural sensitivity and intercultural communication competence.

Fritz et al. (2001) tested ISS with a sample of 541 German students of business administration. Confirmatory factor analysis showed that the scale held satisfactorily. Peng (2006) investigated the intercultural sensitivity of English major students, non-English major students, and multinational employees in China. Results showed different weightings of the 5 elements of intercultural sensitivity of the 3 subsamples. Hu (2011) measured the IS level of 175 undergraduates in Shanxi University in China and found that the mean value of respect of cultural difference was the highest. Soltani (2014) studied the effect of ethnic background on Iranian EFL learners’ intercultural sensitivity and found a strong relationship between them. Zhang (2015) surveyed intercultural sensitivity and English-cultural-loaded vocabulary acquisition of 500 non-English major undergraduate students and found a positive correlation between them. Nameni and Dowlatabadi (2019) investigated the intercultural communicative competence and intercultural sensitivity of 400 medical students in Iran based on ethnic differences and found that the four ethnic groups had moderate levels of ICC and IS. Zhang and Han (2019) measured and compared the intercultural sensitivity of American students from a mid-sized state university in the south and a small-sized private liberal arts school in the mid-west, and found that students from the mid-sized state university were more interculturally sensitive than their counterparts in two dimensions of the Intercultural Sensitivity Scale (ISS). Loebel et al. (2021) adapted and validated ISS in a sample of university students from Chile and verified the factor structure of the scale.

In addition to the above research, the previous study has identified the influencing factors of intercultural sensitivity, such as gender (Holm et al., 2009; Petrović and Zlatković, 2009; Segura-Robles and Parra-González, 2019), overseas experience (traveling/studying/exchange program; Burdette-Williamson, 1997; Olson and Kroeger, 2001; Straffon, 2003; Engle and Engle, 2004; Williams, 2005; Gordon and Mwavita, 2018; Yurur et al., 2018), and foreign language proficiency (Burdette-Williamson, 1997; Olson and Kroeger, 2001; Paige et al., 2003; Engle and Engle, 2004).

Though the intercultural sensitivity of university students in many countries and areas has been well investigated, the research on intercultural sensitivity level of university students in Macao has yet to be made.

### 1.2. Research question

The present study intends to enrich the existing literature about the empirical research on intercultural sensitivity by measuring the IS level of university students in Macao and by exploring whether there are any significant differences in student groups in terms of gender, grade, program (language of construction adopted a major), courses related to intercultural communication (IC-related courses), overseas experience, and foreign language proficiency test.

To address the objectives of the present study, the following research questions were posed:

1. What is the intercultural sensitivity level of university students in the multicultural city Macao?
2. Concerning the IS levels of university students in Macao, are there any significant differences in student groups in terms of gender, IC-related courses, grade, program, overseas experience, and foreign language proficiency?

## 2. Methods

In this research, the quantitative method was adopted as this method has two significant advantages. First, it can be administered and evaluated quickly. There is no need to spend time at the organization prior to administering the survey, and the responses can be tabulated in a short time. Second, numerical data obtained through this approach facilitates comparisons between groups, as well as allowing determination of the extent of agreement or disagreement between respondents (Yauch and Steudel, 2003).

### 2.1. Sampling method

In this research, convenience sampling method was adopted to collect data. Convenience sampling method is a type of non-probability or nonrandom sampling in which members of the target population are selected for the purpose of the study if they meet certain practical criteria, such as geographical proximity, availability at a certain time, easy accessibility, or the willingness to volunteer (Dörnyei, 2007). Furthermore, this sampling strategy is favored in order to obtain an in-depth description of a focused individual in a population (Creswell et al., 2013). “Captive audiences, such as students, in the researchers’ own institution are prime examples of convenience sampling” (Dörnyei, 2007). As the respondents of this research are university students from the first author’s own university, they are conveniently available subjects and the survey is easy to operate, the convenience sampling method is the best fit for the purpose of this research.

### 2.2. Participants

A sample of 375 Macao university students from City University of Macau was selected through a convenient sampling method. Among the participants, there were 130 males (34.7%) and 245 females (65.3%). They came from four grades, including 63 freshmen, 157 sophomores, 82 junior students, and 73 senior students. As for the programs, 94 students came from the Chinese program, 250 students came from the English program, and 31 students came from the Portuguese program.

### 2.3. Instruments

To reach the research objective, two measurement instruments have been adopted.

One instrument is the Intercultural Sensitivity Scale (ISS) developed by Chen and Starosta (2000), as shown in Table 1. ISS is a 5-point Likert-type scale (1 = totally disagree; 2 = disagree; 3 = neutral; 4 = agree; 5 = totally agree). No changes have been made to the items in the scale. Negative questions were reverse-scored and recoded to calculate the mean value. This instrument was used to answer research question 1.

**Table 1:** The Intercultural Sensitivity Scale [ISS, (Chen and Starosta, 2000)].

Dimensions	Items
Intercultural engagement	1. I enjoy interacting with people from different cultures. 11. I tend to wait before forming an impression of culturally-distinct counterparts. 13. I am open-minded to people from different cultures. 21. I often give positive responses to my culturally-different counterpart during our interaction. 22. I avoid those situations where I will have to deal with culturally-distinct persons. 23. I often show my culturally-distinct counterpart my understanding through verbal or nonverbal cues. 24. I have a feeling of enjoyment toward differences between my culturally-distinct counterpart and me.
Respect of cultural difference	2. I think people from other cultures are narrow-minded. 7. I do not like to be with people from different cultures. 8. I respect the values of people from different cultures. 16. I respect the ways people from different cultures behave. 18. I would not accept the opinions of people from different cultures. 20. I think my culture is better than other cultures.
Interaction confidence	3. I am pretty sure of myself in interacting with people from different cultures. 4. I find it very hard to talk in front of people from different cultures. 5. I always know what to say when interacting with people from different cultures. 6. I can be as sociable as I want to be when interacting with people from different cultures. 10. I feel confident when interacting with people from different cultures.
Interaction enjoyment	9. I get upset easily when interacting with people from different cultures. 12. I often get discouraged when I am with people from different cultures. 15. I often feel useless when interacting with people from different cultures.
Interaction attentiveness	14. I am very observant when interacting with people from different cultures. 17. I try to obtain as much information as I can when interacting with people from different cultures. 19. I am sensitive to my culturally-distinct counterpart’s subtle meanings during our interaction.

Another instrument was designed by the authors. It is a sociodemographic questionnaire concerning gender, grade, program, IC-related courses, overseas experience, the language proficiency test, and ways of multicultural contacts among university students in Macao. This instrument was used to answer research question 2.

**Figure 1:**
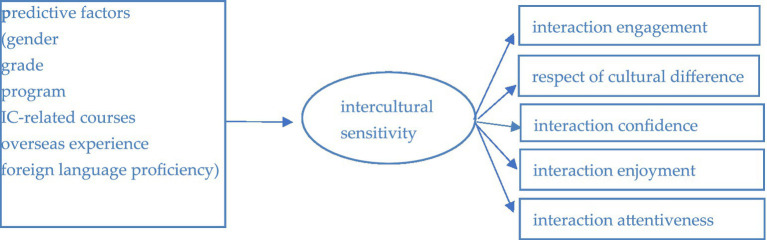
The research model.

The respondents were asked to answer the questions in the two questionnaires at one time (Figure 1).

### 2.4. Procedures

To check semantic coherence, the ISS went through the forward-backward-forward translation technique. The back-translated version of the scale was reviewed by monolinguals of both English and Chinese to discern possible errors that could influence the understanding of the translated instrument.

Before the survey, informative meetings were held with the students, the purpose of this research and the ethical principles of the project were addressed to them and they were informed that the survey would be anonymous, and the data would be for academic research purposes only. Students who agreed to participate in the study filled out the Chinese version of ISS through Tencent Questionnaire voluntarily and anonymously. In total, 385 copies of a questionnaire were distributed from July 1st to 8th, 2022. Subsequently, 380 were collected and 375 were analyzed after excluding 5 invalid ones (with incomplete data).

### 2.5. Data analysis method

Data management and analysis were performed with SPSS25.0. Descriptive analysis, frequency, and percentage were used for an overall description of the participants. The mean value of each subscale was measured. Mean values of 1–2.5 were interpreted as “low,” 2.5–3.5 as “moderate,” and 3.5–5 as “high” levels of IS (Nameni and Dowlatabadi, 2019).

The samples used for comparison in this study did not meet the classic requirements of parametric tests—normality (data have a normal distribution), homogeneity of variances (groups have the same variance), and independence (whether two variables or attributes are independent). As non-parametric studies have the same statistical rigor as parametric studies (Mumby, 2002), two kinds of nonparametric tests, including the Mann–Whitney *U* test and the Kruskal-Wallis *H* test, were carried out in this study. The Mann–Whitney *U* test, which is known as the Wilcoxon rank-sum test, tests for differences between two groups on a single, ordinal variable with no specific distribution. When data do not meet the parametric assumptions of the *t*-test, the Mann–Whitney *U* tends to be more appropriate (McKnight and Najab, 2010). The Kruskal-Wallis is a nonparametric statistical test that assesses the differences among three or more independently sampled groups on a single, non-normally distributed continuous variable. It is a more generalized form of the Mann–Whitney *U* test and is the nonparametric version of the one-way ANOVA (McKnight and Najab, 2010).

## 3. Results

### 3.1. Reliability analysis

In this research, different analyses, including Cronbach’s Alpha (*α*), Composite Reliability (CR), and Average Extracted Variance (AVE), were carried out to verify the reliability of the survey. The results showed that the reliability indices were acceptable, and as the values of Cronbach’s alpha (*α*) and composite reliability (CR) were above 0.70, and the values of average variance extracted (AVE) were higher than 0.50, and there is no problem with the convergent validity. The results of the reliability analysis are shown in Table 2.

**Table 2:** Reliability and validity indices.

Dimensions	Alpha (*α*)	CR^*^	AVE^**^
Interaction engagement	0.836	0.8463	0.5277
Respect of cultural difference	0.916	0.9171	0.8469
Interaction confidence	0.859	0.8625	0.6771
Interaction enjoyment	0.894	0.8949	0.7398
Interaction attentiveness	0.869	0.8642	0.7613

*Composite Reliability, **Average Extracted Variance.

### 3.2. Descriptive analyses

Descriptive data were generated for all variables. The results of the descriptive analyses are shown in Table 3. What is striking about the data in this table is that interaction engagement, respect of cultural difference, interaction enjoyment, and interaction attentiveness have high mean values (>3.5), while the mean value for interaction confidence (3.1) is on moderate level.

**Table 3:** Descriptive analyses of each dimension.

Dimensions	Minimum	Maximum	Mean	Std. Deviation
Interaction engagement	1.00	5.00	4.1696	0.66351
Respect of cultural difference	1.00	5.00	4.3307	0.76783
Interaction confidence	1.00	5.00	3.1076	0.81248
Interaction enjoyment	1.00	5.00	3.6320	0.94955
Interaction attentiveness	1.00	5.00	4.0347	0.76966

### 3.3. Inferential analyses

The first inferential analysis (as in Table 4) was carried out on the difference between males and females. In the results in terms of gender, there was a significant difference in interaction confidence, with *p* = 0.000. In this case, the male students obtained a higher mean rank (MR = 223.65) than that of the female students (MR = 169.08).

**Table 4:** Mann–Whitney *U* test for different dimensions according to gender.

Dimension	Gender	Mean Rank	*U*	*Z*	*P*
Interaction engagement	Female	190.73	15,255.000	−0.676	0.499
	Male	182.85			
Respect of cultural differences	Female	195.14	14,175.000	−1.869	0.062
	Male	174.54			
Interaction confidence	Female	169.08	11,290.500	−4.709	0.000
	Male	223.65			
Interaction enjoyment	Female	195.39	14,113.500	−1.831	0.067
	Male	174.07			
Interaction attentiveness	Female	184.00	14,945.000	−1.012	0.311
	Male	195.54			

The second inferential analysis (as in Table 5) was made on the differences between the students who have taken the IC-related courses and those who have not. Among the participants, 267 (71.2%) students have studied the courses related to intercultural communication, and 108 (28.8%) students have not. Mann–Whitney *U* test reflected that there were significant differences in interaction confidence (*p* = 0.006) and interaction attentiveness (*p* = 0.026). Concerning interaction confidence, the students who have taken the courses related to intercultural communication obtained a higher mean rank value (MR = 193.77) than those who have not (MR = 173.73) and for interaction attentiveness, the students who have taken the courses related to intercultural communication obtained a higher mean rank value (MR = 197.57) than those who have not (MR = 164.35).

**Table 5:** Mann–Whitney *U* test for different dimensions according to whether one has taken the IC-related courses.

Dimension	IC-related Courses	Mean Rank	*U*	*Z*	*p*
Interaction engagement	Yes (267)	194.47	12,689.500	−1.833	0.067
	No (108)	172.00			
Respect of cultural differences	Yes (267)	189.12	12,876.500	−1.731	0.084
	No (108)	185.22			
Interaction confidence	Yes (267)	193.77	11,864.000	−2.728	0.006
	No (108)	173.73			
Interaction enjoyment	Yes (267)	195.68	14,118.000	−0.319	0.750
	No (108)	169.01			
Interaction attentiveness	Yes (267)	197.57	12,367.500	−2.226	0.026
	No (108)	164.35			

The analysis on the differences among the students according to the grades is shown in Table 6. The Kruskal-Wallis *H* test indicated that there was a significant difference in terms of interaction enjoyment, with *p* = 0.003. The most surprising aspect of the data is that the mean rank of the freshmen (MR = 233.52) was the highest among the four grades.

**Table 6:** Kruskal-Wallis *H* test for different dimensions according to grades.

Dimension	Grade	Mean Rank	*H*	df	*p*
Interaction engagement	Freshmen	220.29	7.4393	3	0.059
	Sophomores	185.44			
	Juniors	173.95			
	Seniors	181.43			
Respect of cultural differences	Freshmen	187.92	0.658	3	0.883
	Sophomores	187.51			
	Juniors	194.74			
	Seniors	181.56			
Interaction confidence	Freshmen	175.56	1.384	3	0.709
	Sophomores	188.03			
	Juniors	189.55			
	Seniors	196.93			
Interaction enjoyment	Freshmen	233.52	13.811	3	0.003
	Sophomores	180.86			
	Juniors	174.43			
	Seniors	179.32			
Interaction attentiveness	Freshmen	184.73	1.398	3	0.706
	Sophomores	182.03			
	Juniors	193.34			
	Seniors	197.66			

Table 7 shows the results on the differences among student groups in terms of program. In higher education in Macao, there are three programs: Chinese, English, and Portuguese. Among the participants, 94 students majored in Chinese program, 250 students majored in English program, and 31 students majored in Portuguese program. The data reflected that no significant difference was found among the participants of the three programs.

**Table 7:** Kruskal-Wallis *H* test for different dimensions according to program (language of instruction adopted in one’s major).

Dimension	Program	Mean Rank	*H*	df	*p*
Interaction engagement	Chinese program	179.3	2.930	2	0.231
	English program	194.19			
	Portuguese program	164.45			
Respect of cultural differences	Chinese program	182.74	3.562	2	0.168
	English program	193.59			
	Portuguese program	158.85			
Interaction confidence	Chinese program	190.35	0.532	2	0.766
	English program	188.76			
	Portuguese program	174.79			
Interaction enjoyment	Chinese program	183.43	1.080	2	0.583
	English program	187.45			
	Portuguese program	206.32			
Interaction attentiveness	Chinese program	186.96	4.309	2	0.116
	English program	192.92			
	Portuguese program	151.45			

Of the 375 students who completed the questionnaire, as for overseas experiences (including study abroad, study tour, internship, traveling, etc.), 101 of them (27%) had no experience abroad, 146 of them (39%) had 1–2 overseas experiences, and128 of them (34%) had been abroad 3 or more times. The data in Table 8 indicated that no significant difference was found among the students with different overseas experiences.

**Table 8:** Kruskal-Wallis *H* test for different dimensions according to overseas experience.

Dimension	Overseas experience	Mean Rank	*H*	df	*p*
Interaction engagement	0	177.13	1.53	2	0.465
	01-February	194.1			
	≥3	189.63			
Respect of cultural differences	0	177.83	1.588	2	0.452
	01-February	189.16			
	≥3	194.7			
Interaction confidence	0	176.09	1.763	2	0.414
	01-February	193.64			
	≥3	190.96			
Interaction enjoyment	0	173.5	2.923	2	0.232
	01-February	189.49			
	≥3	197.74			
	
Interaction attentiveness	0	182.23	0.446	2	0.8
	01-February	191.15			
	≥3	188.95			

The results obtained from the inferential analysis on the differences between the students who have passed a foreign language proficiency test and those who have not are presented in Table 9. The foreign language proficiency tests include IELTS, TOEFL, TOEIC, College English Test Band 4, College English Test Band 6, Public English Test, China Accreditation Test for Translators and Interpreters, Business English Certificate, and other foreign language proficiency tests (such as Test for Proficiency in Korean, the Japanese-Language Proficiency Test), etc. It is shown that 242 (64.5%) participants have passed a foreign language proficiency test, and 133 (35.5%) have not. There was a significant difference between the students who have passed a language proficiency test and those who have not in terms of interaction confidence, with *p* = 0.017 and the mean rank of those who have passed a foreign language proficiency test (MR = 197.77) was higher than that of those who have not (MR = 170. 23).

**Table 9:** Mann–Whitney *U* test for different dimensions according to foreign language proficiency test.

Dimension	Foreign language proficiency test	Mean Rank	*U*	*Z*	*P*
Interaction engagement	Yes	191.12	15,337.500	−0.758	0.448
	No	182.32			
Respect of cultural differences	Yes	194.33	14,560.500	−1.629	0.103
	No	176.48			
Interaction confidence	Yes	197.77	13,729.000	−2.390	0.017
	No	170.23			
Interaction enjoyment	Yes	186.13	15,639.500	−0.456	0.648
	No	191.41			
Interaction attentiveness	Yes	191.34	15,285.000	−0.830	0.406
	no	181.92			

## 4. Discussion and conclusion

The present study aimed to investigate the IS level of Macao university students and explore whether there were any significant differences in student groups in terms of gender, grade, program, courses related to intercultural communication, overseas experience, and foreign language proficiency. The research data showed that the university students in Macao had a relatively high IS level: Among the five dimensions, three dimensions, including interaction engagement, respect of cultural difference, and interaction attentiveness, had mean values which were higher than 4. The mean value of respect of cultural difference was the highest (4.3307), and that of interaction confidence (3.1076) was the lowest. A possible explanation for the relatively high level of intercultural sensitivity of university students in Macao might be the following:

First, multicultural context contributes significantly to students’ high level of intercultural sensitivity, which is consistent with previous studies (Roh, 2014; Segura-Robles and Parra-González, 2019). University students in Macao have been surrounded by a uniquely multicultural environment. Walking on the streets of Macao, they can see the bi-lingual or tri-lingual street signs all over the city. Getting on the buses, they can hear Chinese, Cantonese, Portuguese, and English adopted in the automatic stop announcer. They meet teachers, students, and even working staff of different cultural background. Further, they can visit the multicultural places of interest in Macao and take part in the international events hold here. For example, just from October to November 2022, there were a series events held in Macao, to name a few, the 34th Macao International Music Festival, the 4th “Encounter in Macao— Arts and Cultural Festival between China and Portuguese-speaking Countries,” the 25th Lusofonia Festival (featuring the “China and Portuguese-speaking Countries Film Festival,” the “Traditional Music and Dance Performance in the Community” and the “Chinese and Portuguese Picture Book Fair”), the 22nd Macau Food Festival, and the 69th Macau Grand Prix. Having been immersed in a fascinating heterogeneous city of different cultures, university students in Macao can not only perceive, understand, accept, and respect cultural differences, but also engage in intercultural communication more actively.

Second, there are many other opportunities for university students in Macao to have direct and indirect intercultural communication contact as indicated in demographic information. For indirect intercultural contacts, in addition to taking the compulsory foreign language courses, 267 (71.2%) of the participants had taken courses related to intercultural communication, and 350 (93%) of them read foreign literary classics, watched foreign movies and TV programs, or listened to foreign music and for direct intercultural contacts, 274 (73%) of the students had overseas experience, 258 (68.8%) of them traveled abroad, and 128 (34%) of them participated in international exchange programs. Furthermore, among these participants, 258 (68.8%) of them have passed a language proficiency test (IELTS, TOFEL, CET-4, CET-6, etc.) According to Yurur et al. (2018), exposure to other cultures by participating previously in student exchange programs, work, and travel programs, and spending a long period of time abroad increased people’s intercultural sensitivity. All the participants in this research were not lacking an opportunity to have direct interaction with people from different cultures in Macao. With knowledge about intercultural communication, immersing in a multi-cultural harmonious environment, various intercultural contacts enabled them to discern, understand, and respect cultural differences. The findings demonstrated that most of them did not consider their own culture better than others, but respected values and behaviors of people from other cultures. They did not resist interacting with people from different cultures, and blindly reject the opinions of people from different cultures. They engaged in intercultural communication actively, focused on the intercultural process and effects, and got enjoyment in intercultural communication.

However, in the current study, the mean value of the interaction confidence of the university students in Macao was not very high. This result is similar to the research of certain previous studies (Hua, 2020). There are several possible explanations for it. First, the result is related to the language proficiency of the participants, because limited language proficiency is identified as a major obstacle in developing social skills and confidence (Khawaja and Stallman, 2011) and according to Clement’s linguistic confidence theory, due to limited contact before immersion, participants cannot have sufficient linguistic confidence, hence their interaction confidence cannot be built up (Wong, 2015). In this survey, 35.5% of the participants have not passed a language proficiency test. Furthermore, research data of this study (as in Table 9) indicated that the mean rank of those who have passed a foreign language proficiency test was higher than that of those who have not. Second, another possible explanation for this is lack of real-life intercultural communication practice. Precisely, social distancing and lockdown during the COVID-19 pandemic period decreased the quantity and quality of real-life communication which influenced the interaction confidence of the participants. Confidence in intercultural communication comes from communication behavior. In the developmental nature of intercultural sensitivity, the more frequently one communicates, the more confident one becomes (Bennett, 1986). Third, this result is also likely related to personality. Research on Chinese students’ perceived barriers to effective intercultural communication (Zhang and Zhou, 2021) found that two major personality-related barriers, including shyness and lack of confidence, influenced the participants’ performance in intercultural communication.

The demographic information indicated certain specific characteristics of the intercultural sensitivity level of university students in Macao. Concerning the relationship between gender and the intercultural sensitivity level, the current study found that there was a significant difference in interaction confidence. The male students showed a higher mean rank than that of the female students. This result differs from the previous study results made by Kim and Mo (2011) and Mellizo (2017), but it is broadly consistent with the findings of Meydanlioglu et al. (2015) and Boudouaia et al. (2022). Given that scholars have got exact opposite results, it is difficult to explain this result in this research; a further study with more focus on interaction confidence concerning gender is therefore suggested.

As for the relationship between IC-related courses taken by the university students in Macao and the IS level of them, the results of this study showed that there were significant differences in interaction confidence and interaction attentiveness. The students who have taken the IC-related courses showed more interaction confidence and higher interaction attentiveness. This research result reflected the effectiveness of setting IC-related courses in enhancing IS level of university students. The findings showed consistency with previous literature, which argued that it is necessary to emphasize the formulation of more IC-related courses (Liu et al., 2022).

Regarding the IS level of students from different grades, there was a comparison among students from four grades. This study found that there was a significant difference among the students in terms of interaction enjoyment and the mean rank of the freshmen was the highest. A probable explanation for this surprising result is that all the freshmen in this survey were language majors. This result is consistent with the findings of previous studies on non-language majors and language majors. Wei (2019) made research on the intercultural sensitivity of non-English major freshmen and found that the mean rank of interaction enjoyment of the participants was the lowest. The barriers of the non-English majors’ intercultural communication were summarized as the following: the deficiency of communication skills, the deficiency of culture knowledge, the deficiency of experience, and the deficiency of confidence. In addition, Wattanavorakijkul (2020) found that English majors had more exposure to using English and communicating with foreigners than participants in other disciplines. Their English proficiency enabled them to enjoy intercultural experiences and interact more with friends from different cultures. The language major freshmen of this research studied language skills of listening, speaking, reading, writing, and translation every day. They were accustomed to speaking in English or Portuguese, and did not have obvious language barriers in communication, hence had more interaction enjoyment than other participants.

In higher education in Macao, there are three programs (languages of instruction adopted in one’s major): Chinese, English, and Portuguese. The data reflected that no significant difference was found among the participants of the three programs. There are several possible explanations for this result. First, the sources of students for these three programs are similar, mainly students from mainland China and local students from Macao. Second, most of the teachers in each faculty have international education background. Third, all the students are in the same cultural atmosphere.

The data of this study indicated that there was no significant difference between those who have been abroad and those who have not. With respect to the relationship between the overseas experience and one’s level of intercultural sensitivity, the findings of different academic research results were controversial. Some researchers (Anderson et al., 2006; Yashima, 2010) provided evidence that participants with short-term overseas experience exhibit greater improvement in intercultural sensitivity than those without, and some (Kirillova et al., 2015) declared that short-term overseas experience (such as volunteer tourism) has the potential to simultaneously promote and inhibit intercultural understanding. In this study, as the contact quality and contact quantity of the students’ overseas experience have not been investigated, and whether the multicultural academic environment has enhanced the IS level of the students was not clear. As a result, the reasons behind this finding require further research.

Finally, as for the relationship between the foreign language proficiency test and the IS level of the students, there was a significant difference in terms of interaction confidence. Those who have passed a foreign language proficiency test had higher IS level than those who have not. This research result is like that of Iqbal (2019) which put forward that foreign language proficiency is associated with decreased fear of language barriers.

Intercultural sensitivity has been regarded as a very important ability necessary for living harmoniously in today’s pluralistic world. It is not an instinctive and universal aspect of human behavior. This study examined the IS level of university students from Macao and enriched the existing literature about the empirical research on intercultural sensitivity. It has been indicated that intercultural sensitivity is a developmental process, and cultural environment and training are two important points in the development of high levels of intercultural sensitivity. The results indicated the influence of intercultural experience, including physical experience and indirect exposures on IS levels of university students in Macao. The findings of this study are especially useful to education policymakers and educators in identifying the strengths and weaknesses of their university students in intercultural sensitivity. For Macao, Education and Youth Development Bureau of Macao (DSEDJ) can put forward policies that are more conducive to enhancing the intercultural sensitivity level of Macao university students and university teachers can arrange their classroom teaching and extracurricular activities more scientifically and efficiently. Finally, the intercultural sensitivity of the college students can be further improved.

## 5. Limitations and future research

This study has several limitations and elicits suggestions for future research on intercultural sensitivity. First, there is a limitation on generalizing these results to the entire Macao undergraduates, since the sampling was based on students in City University of Macau. Second, the ISS scale adopted in this study was European-centric. It is necessary to consider whether the results based on the instrument scale could be an adequate reflection of the characteristics of the intercultural sensitivity among Macao university students. Third, qualitative method is adopted in this research, but it is limited to a self-report survey. An explanatory sequential mixed method, including self-report survey, knowledge-based test, and interview, could be adopted in future research.

## Data availability statement

The original contributions presented in the study are included in the article/supplementary materials, further inquiries can be directed to the corresponding author.

## Ethics statement

The studies involving human participants were reviewed and approved by the Ethics Committee of City University of Macau. The patients/participants provided their written informed consent to participate in this study.

## Author contributions

HC contributed to the conception, design of the study, and wrote the first draft of the manuscript. HC and BH organized the database and performed the statistical analysis. All authors contributed to the manuscript revision, read, and approved the submitted version.

## Funding

This research was funded by Macao Foundation, grant number MF2033.

## Conflict of interest

The authors declare that the research was conducted in the absence of any commercial or financial relationships that could be construed as a potential conflict of interest.

## Publisher’s note

All claims expressed in this article are solely those of the authors and do not necessarily represent those of their affiliated organizations, or those of the publisher, the editors and the reviewers. Any product that may be evaluated in this article, or claim that may be made by its manufacturer, is not guaranteed or endorsed by the publisher.
